# Role of Mitochondrial RNA Polymerase in the Toxicity of Nucleotide Inhibitors of Hepatitis C Virus

**DOI:** 10.1128/AAC.01922-15

**Published:** 2016-01-29

**Authors:** Joy Y. Feng, Yili Xu, Ona Barauskas, Jason K. Perry, Shekeba Ahmadyar, George Stepan, Helen Yu, Darius Babusis, Yeojin Park, Krista McCutcheon, Michel Perron, Brian E. Schultz, Roman Sakowicz, Adrian S. Ray

**Affiliations:** Gilead Sciences, Foster City, California, USA

## Abstract

Toxicity has emerged during the clinical development of many but not all nucleotide inhibitors (NI) of hepatitis C virus (HCV). To better understand the mechanism for adverse events, clinically relevant HCV NI were characterized in biochemical and cellular assays, including assays of decreased viability in multiple cell lines and primary cells, interaction with human DNA and RNA polymerases, and inhibition of mitochondrial protein synthesis and respiration. NI that were incorporated by the mitochondrial RNA polymerase (PolRMT) inhibited mitochondrial protein synthesis and showed a corresponding decrease in mitochondrial oxygen consumption in cells. The nucleoside released by the prodrug balapiravir (R1626), 4′-azido cytidine, was a highly selective inhibitor of mitochondrial RNA transcription. The nucleotide prodrug of 2′-*C*-methyl guanosine, BMS-986094, showed a primary effect on mitochondrial function at submicromolar concentrations, followed by general cytotoxicity. In contrast, NI containing multiple ribose modifications, including the active forms of mericitabine and sofosbuvir, were poor substrates for PolRMT and did not show mitochondrial toxicity in cells. In general, these studies identified the prostate cell line PC-3 as more than an order of magnitude more sensitive to mitochondrial toxicity than the commonly used HepG2 cells. In conclusion, analogous to the role of mitochondrial DNA polymerase gamma in toxicity caused by some 2′-deoxynucleotide analogs, there is an association between HCV NI that interact with PolRMT and the observation of adverse events. More broadly applied, the sensitive methods for detecting mitochondrial toxicity described here may help in the identification of mitochondrial toxicity prior to clinical testing.

## INTRODUCTION

Hepatitis C virus (HCV) is a major cause of liver disease worldwide and is the leading reason for liver transplantation in North America and Europe (1). In 2011, the treatment of chronic HCV infection was advanced with the regulatory approval of two protease inhibitors, telaprevir and boceprevir, which directly targeted the virus and increased sustained viral response rates (2). However, these agents were given in combination with pegylated interferon (IFN) and ribavirin (RBV), adding new side effects on top of the already challenging tolerability profile of the prior standard of care. These combinations also had complicated dosing regimens, were only effective in patients with genotype 1 infection, and were less efficacious in many of the populations most in need of therapy (3). These limitations led to the continued pursuit of agents to treat HCV infection that exhibit greater efficacy and improved tolerability and more broadly address the needs of those afflicted with HCV infection, including patients around the world infected with other HCV genotypes and those with advanced liver disease.

**FIG 1:**
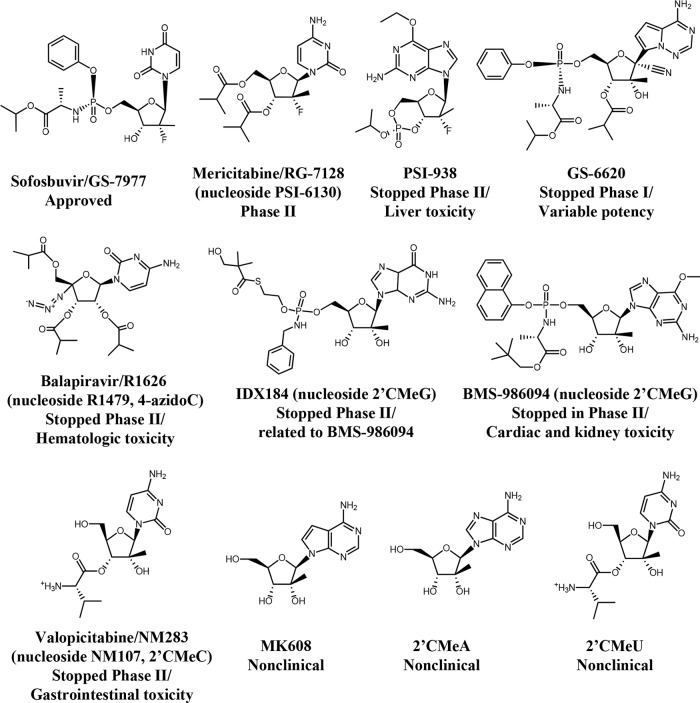
Structures of HCV NI and their clinical progress. The names of some corresponding nucleosides for the prodrugs are provided in parentheses.

In particular, nucleotide inhibitors (NI) of HCV RNA synthesis that serve as alternate substrates and inhibitors of the viral RNA-dependent RNA polymerase (HCV nonstructural protein 5B [NS5B]) have garnered substantial attention. The binding of NI to the highly conserved NS5B active site results in activity that is maintained across genotypes and substantial loss of viral fitness upon the infrequent development of resistance mutations (4, 5). The promising attributes of NI proved difficult to translate into clinical success until the recent approval of sofosbuvir in 2013. Failure during the clinical development of NI candidates has been primarily due to toxicity. The first two nucleoside analogs to enter clinical development, valopicitabine (NM283; prodrug of 2′-*C*-methyl (2′CMe) cytidine [2′CMeC, NM107] and balapiravir (R1626; prodrug of 4′-azido cytidine [4′-azidoC, R1479] (Fig. 1) had their development programs halted during phase 2 studies due to the observation of gastrointestinal and hematologic toxicity, respectively (6). Development of PSI-938 (prodrug of 2′F,2′CMe guanosine) was discontinued due to the observation of hepatic toxicity in phase 2b (7). IDX184 and BMS-986094, prodrugs that deliver the same pharmacologically active triphosphate (TP) (2′CMeGTP), had their clinical programs halted following the observation of cardiac and kidney toxicity with BMS-986094 (8, 9). Most recently, VX-135, a uridine analog with an undisclosed structure, was placed on partial clinical hold based on the observation of elevated liver enzymes (10). Other NI have likely never reached clinical trials due to the observation of toxicity during preclinical studies. For example, MK608 showed promising antiviral activity in chimpanzees (11), but further clinical studies have not been reported to date.

The mechanisms for toxicity of ribonucleotide analogs have not been well characterized but could theoretically arise from incorporation into cellular RNA by human RNA polymerases I, II, III (PolI, PolII, and PolIII) and the mitochondrial RNA polymerase (PolRMT). In comparison to PolII, PolRMT has been shown to be particularly vulnerable to inhibition by ribonucleotide analogs due to the lack of a functional proofreading activity (12). However, neither PolI nor PolIII has been studied as a source of cytotoxicity for this class of compounds. In this study, we assessed the potential for clinical HCV NI (Fig. 1) to cause toxicity in various functional cell-based assays, including assays for (i) cytotoxicity in multiple human cell lines and primary cells, (ii) reductions in mitochondrial- and nuclear-DNA-encoded proteins, and (iii) changes in mitochondrial oxygen consumption. The interactions of NI with purified human DNA and RNA polymerases in biochemical assays were also assessed. While general cytotoxicity or the inhibition of the human DNA polymerases or RNA PolI, PolII, and PolIII did not consistently identify NI associated with clinical toxicity, interactions with PolRMT in biochemical assays coupled with corresponding decreases in mitochondrial protein production and cellular respiration suggest this polymerase as an important determinant of toxicity. A screening paradigm is proposed that will aid in identifying the potential for mitochondrial toxicity of nucleotide analogs that can also be applied more broadly to other classes of drugs.

## MATERIALS AND METHODS

### Reagents.

All NI were synthesized by Gilead Sciences, Inc. Puromycin, 5-flurouracil (5-FU), and alpha-amanitin were purchased from Sigma-Aldrich (St. Louis, MO). All radioactively labeled nucleoside triphosphates (NTPs) were purchased from PerkinElmer (Shelton, CT).

### Cell culture and cytotoxicity studies.

The following cell lines were obtained from the indicated sources: Huh-7 (differentiated hepatocellular carcinoma; JCRB Cell Bank, Japan), HepG2 (hepatoblastoma; ATCC), PC-3 (prostate metastatic carcinoma; ATCC), MRC5 (fibroblast from normal lung tissue; ATCC), and MT-4 (human T-cell leukemia virus 1 [HTLV-1]-transformed human T lymphoblastoid cells; NIH AIDS Research and Reference Reagent Program). Primary human hepatocytes were from Bioreclamation IVT (Westbury, NY) or Life Technologies (Carlsbad, CA). Human peripheral blood mononuclear cells (PBMCs) were isolated from human buffy coats obtained from healthy volunteers (Stanford Blood Bank, Palo Alto, CA) using standard Ficoll separation and stimulated as described elsewhere (13) and were tested at both quiescent and stimulated stages. Quiescent PBMCs were stimulated with 10 units per ml of recombinant human interleukin 2 (hIL-2) and 1 μg per ml phytohemagglutinin P (PHA-P) for 48 h prior to drug treatment. Normal human primary bone marrow (BM) light-density cells were from three different lots obtained from AllCells (Emeryville, CA) or Lonza (Walkersville, MD). Primary rat neonatal cardiomyocytes were isolated from 3-day-old Sprague Dawley rat pups as described previously (14).

All cells were cultured at 37°C in a 5% CO_2_ incubator with 90% humidity unless noted otherwise. Detailed culture conditions for each cell line and primary cell can be found in the supplemental material. All cells were treated with the various compounds for 5 days, except for human erythroid and myeloid progenitors, which were treated for 14 days. After the incubation period, cell viability was measured by the addition of CellTiter Glo viability reagents (Promega, Madison, WI). The luminescence signal was quantified on an Envision luminescence plate reader (PerkinElmer, Waltham, MA) after incubation of the reagents and cells for 10 min at room temperature. The compound concentration that caused a 50% decrease in the luminescence signal (CC_50_), a measure of toxicity, was calculated by nonlinear regression using a sigmoidal dose-response (variable slope) equation, as follows:

1 Y=bottom+(top−bottom)/{1+10^[(LogCC50−X)×hillslope]}

where *X* is the log of the concentration of the test compound, *Y* is the response, and the bottom and top values were fixed at 0 and 100, respectively, unless a significant variation was noted. CC_50_ values were calculated as the average of the results of three or four independent experiments.

The effects of the compounds on the proliferation of human erythroid and myeloid progenitors were tested in MethoCult84434, a methylcellulose-based colony assay conducted by Stemcell Technology (Vancouver, Canada) (15). After a 14-day culture, hematopoietic progenitor colonies (CFU-E, BFU-E, CFU-GM, and CFU-GEMM [E, erythroid; BFU, burst-forming unit; GM, granulocyte/macrophage; GEMM, multilineage progenitors]) were enumerated and the CC_50_ values were calculated using equation 1.

### Measurement of active triphosphate metabolite in PC-3 cells.

The amount of nucleoside triphosphate was determined from three independent studies by ion-pairing liquid chromatography-tandem mass spectrometry (LC-MS/MS) as previously described (16). All compounds were incubated with the cells at 10 μM, except for BMS-986094 and NM107, which were incubated at 0.1 and 1 μM, respectively, due to the cytotoxicity observed at higher concentrations. Triphosphate concentrations for BMS-986094 and 2′CMeC were applied at doses normalized to the concentrations anticipated after a 10 μM incubation for comparison with the other NI. The reported TP levels are the maximal concentrations observed after 48 h during a 5-day incubation.

### DNA and RNA templates and primers.

Activated fish sperm DNA was purchased from USB/Affymetrix (Santa Clara, CA) and used as a template for the DNA polymerases alpha, beta, and gamma. The DNA template used in the RNA Pol II assay was a 1,188-bp restriction fragment containing the cytomegalovirus (CMV) immediate early promoter (Promega, Madison, WI). The RNA and DNA oligonucleotides used in the mitochondrial RNA polymerase assay (see Table S1 in the supplemental material) were synthesized and PAGE purified by Thermo Scientific/Dharmacon (Lafayette, CO). RNA primer R12 was 5′-^32^P-phosphorylated with [γ-^32^P]ATP (3,000 Ci/mmol) and T4 kinase (New England BioLabs, Ipswich, MA).

### Enzymatic assays.

Human DNA polymerase alpha, isolated from HeLa cell extracts, was from CHIMERx (Madison, WI). Recombinant human DNA polymerase beta, expressed in E. coli, was a gift from Zucai Suo at The Ohio State University. Recombinant human DNA polymerase gamma (including both the large subunit and the small subunit) was cloned, expressed, and purified from insect cells by Gilead Sciences (Foster City, CA) (13). RNA PolII was purchased as part of the HeLaScribe nuclear extract *in vitro* transcription system kit from Promega (Madison, WI). The recombinant human PolRMT and the transcription factors mitochondrial transcription factor A (mtTFA) and B2 (mtTFB2) were purchased from Enzymax (Lexington, KY). FLAG epitope-tagged RNA PolI and PolIII complexes were isolated from rat NSN1 cells and HeLa cells, respectively (17, 18). The inhibition of DNA polymerases alpha, beta, and gamma, PolRMT, and PolII and the rate of single-nucleotide incorporation by PolRMT have been described previously in detail (12, 13, 19). Inhibition of RNA PolI and PolIII was studied using published methods (20, 21). All concentrations were final unless noted otherwise. For the RNA PolI inhibition assay, a reaction mixture containing 50 mM HEPES (pH 7.9), 20% glycerol, 100 mM KCl, 1 mM dithiothreitol (DTT), 50 μg/ml calf thymus DNA, 4 mM MgCl_2_, 5 or 80 μM UTP (0.25 μl of [5,6-^3^H]UTP, >25 Ci/mmol; PerkinElmer), 1 mM noncompeting NTP, competing NTP (5 μM for GTP, CTP, and UTP and 25 μM for ATP), and various concentrations (0 to 500 μM) of NTP analogs was preincubated at 30°C for 5 min. The reaction was initiated with the addition of 5 μl of FLAG affinity-purified RNA PolI. After incubation at 30°C for 20 min to 1 h, the reactions were terminated by pipetting the mixtures onto DEAE-cellulose discs (DE81; Whatman), and the products analyzed as described previously (20, 21).

The RNA PolIII inhibition assay was conducted in a similar manner, except that the concentrations of the competing NTP were modified to 100 μM for CTP and GTP and 500 μM for ATP to compensate for the low assay signal at lower NTP concentrations.

### Mitochondrial protein synthesis assay.

PC-3 cells were treated with the compounds for 5 days and analyzed with the MitoTox MitoBiogenesis in-cell enzyme-linked immunosorbent assay (ELISA) kit (MitoSciences/Abcam, Eugene, Oregon) as described previously (13). The assay uses quantitative immunocytochemistry to measure the protein levels of nuclear DNA-encoded succinate dehydrogenase (SDH-A; complex II [succinate dehydrogenase]) and mitochondrial DNA-encoded cytochrome *c* oxidase (COX-1; complex IV [cytochrome *c* oxidase]) in cultured cells.

### Measurement of the oxygen consumption rate in PC-3 cells.

Mitochondrial respiration was monitored by measuring the rate of oxygen consumption (OCR) of PC-3 cells after 3-day treatments with the compounds, using a Seahorse extracellular flux analyzer (XF^e^-96) based on published protocols (22–24). The optimal cell seeding was determined by measuring the basal OCR of PC-3 cells that were seeded at different densities of 2.5 × 10^3^, 5 × 10^3^, 7.5 × 10^3^, and 10 × 10^3^ cells/well in XF 96-well plates (Seahorse Bioscience, North Billerica, MA) and incubated with 0.5% dimethyl sulfoxide (DMSO) for 3 days. The seeding density of 5 × 10^3^ cells/well yielded basal OCR values of 80 to 100 pmol/minute (<15% coefficient of variation) and was chosen for future studies. Each reagent in the Mito Stress test kit (Seahorse Biosciences), i.e., ATP synthase inhibitor oligomycin, mitochondrial uncoupler carbonyl cyanide-4-(trifluoromethoxy)phenylhydrazone (FCCP), and a fixed-ratio mixture of the mitochondrial complex I (NADH dehydrogenase) inhibitor rotenone and the complex III (CoQH_2_-cytochrome *c* reductase) inhibitor antimycin A, was titrated. The lowest concentration that reached the maximal effect was chosen for each reagent. The OCR signals were normalized by cell numbers using DNA content determined by Hoechst's stain as described below.

PC-3 cells were seeded at a density of 5 × 10^3^ cells/well in XF 96-well plates and incubated with the compounds for 3 days. On the day of the assay, the cell medium was replaced with XF assay medium (pH 7.4) containing 10 mM glucose and 1 mM freshly prepared pyruvate. Mitochondrial respiration was monitored by measuring OCR on a Seahorse extracellular flux analyzer (XF^e^-96). All concentrations listed are the final concentration after mixing unless noted otherwise. Multiple parameters were measured after the sequential injection of oligomycin (2 μM), FCCP (0.25 μM), and the mixture of rotenone (0.5 μM) and antimycin A (0.5 μM). Spare respiratory capacity was obtained by subtracting the rate of basal respiration from the rate of maximal respiration. The data reported for each treatment are the average of the results from six replicates.

### Detection of cellular DNA level.

After mitochondrial respiration was measured, the XF medium was removed and replaced with 60 to 80 μl of 1× TNE buffer (100 mM Tris base, 10 mM EDTA, and 1.75 M NaCl). The cells were lysed by freezing at −80°C for >2 h. The amount of DNA in each well was determined by staining the cells with Hoechst 33258 stain (Thermo Scientific, Wilmington, DE) and measuring the resulting fluorescence signal with an excitation wavelength of 352 nm and a detection wavelength of 461 nm. In parallel, another set of cells was treated with the compounds for 3 days and the ATP levels were measured using CellTiter Glo viability reagents (Promega, Madison, WI).

### Detection of RNA transcripts using RT-PCR.

PC-3 cells were seeded at a density of 20,000 cells/well in a 96-well black clear-bottom plate (Corning, Tewksbury, MA) in 1× F-12K nutrient mixture, Kaighn's modification, with l-Glu, 10% heat-inactivated fetal bovine serum, and penicillin-streptomycin from Life Technologies (Carlsbad, CA) and grown overnight. CX-5461, a known specific RNA PolI inhibitor, was used as the positive control (25). The growth medium was aspirated and replaced with 200 μl of 100 μM BMS-986094, 5 μM CX-5461, or DMSO control prepared in growth medium. At selected time points (0.5, 1, 4, 8, and 24 h), duplicate wells of the compounds and DMSO control were aspirated, the cells were washed 1× with PBS (pH 7.4), and 100 μl of RNA lysis buffer was added (Promega, Sunnyvale, CA). RNA was purified using the Wizard SV 96 total RNA isolation kit (Promega) and eluted in 100 μl of nuclease-free water. The TaqMan RNA-to-Ct 1-step kit (ABI/Life Technologies) was used for reverse transcription-quantitative PCR (RT-qPCR) amplification and quantification, using the 7900HT Fast real-time PCR system (ABI) under the following conditions: 15 min at 48°C for reverse transcription and 10 min at 95°C for activation, followed by 40 cycles of 15 s at 95°C for denaturation and 1 min at 60°C for annealing and extension. Primer/probe sets included the following: Hs.PT.58.26770695 MYC (IDT PrimeTime standard qPCR assay; GenBank RefSeq accession number NM_002467), Hs.PT.58.264008 MYB (IDT PrimeTime standard qPCR assay; GenBank RefSeq accession number NM_005375), human RNA18S 5 FAM-MGB (Applied Biosystems assay; TaqMan assay identifier [ID] Hs02596862_g1), human mitochondrially encoded ATP synthase 6 (MT-ATP6)–FAM-MGB (Applied Biosystems assay; TaqMan ID Hs02596862_g1), and custom primers for the 5′-external transcribed spacer (ETS) of pre-rRNA (GenBank accession number U13369) (probe, 5′-/56-FAM/TCC GGT ACC/ZEN/CCC AAG GCA C/3IABkFQ/-3′; primer 1, 5′-CAT AAC GGA GGC AGA GAC AG-3′; and primer 2, 5′-AAA AGC CTT CTC TAG CGATCT G-3′ [IDT]). Standard curves for each gene were generated using a pool of the DMSO-treated control RNA diluted serially 1/5 in RNase-free water. Transcripts were quantified relative to the standard curves, and the results expressed as the percentages of the average value of DMSO control wells.

### Computer modeling.

A homology model of PolRMT was constructed based on a ternary structure of T7 RNA polymerase that includes an RNA primer, DNA template, two bound Mg^++^ ions, and an incoming nonhydrolyzable ATP analog (PDB ID 1S76) (26). Although a crystal structure of human POLRMT was recently solved (27), this apo structure is expected to undergo significant conformational changes during transcription, and thus, the T7 RNA polymerase structure was deemed a better starting point. The model was constructed using the Prime package (version 3.0, 2011; Schrodinger, LLC, New York, NY). The overall homology was 27% identity and 44% similarity over residues 336 to 1188 (PolRMT numbering), but the active site was exceptionally well conserved. Only two residues with direct interactions with the substrate differed (T7 RNA polymerase R632 → PolRMT Q992, positioned under the 3′OH of the substrate, and T7 RNA polymerase S541 → PolRMT N926, positioned over the substrate/template base pair). Models of the NI in the PolRMT active site were refined using Macromodel (version 9.9, 2011; Schrodinger, LLC, New York, NY).

## RESULTS

### Evaluation of cytotoxicity in cell lines and primary cells.

The cytotoxicity of a panel of clinical HCV NI was tested in various human cell lines and primary cells for 5 to 14 days (Fig. 1; Tables 1 and 2). Compounds not associated with clinical toxicity generally did not show marked cytotoxicity. For example, sofosbuvir showed modest effects in only one cell line (HepG2), and the nucleoside released by mericatibine, PSI-6130, had no effects on any of the cells at the concentrations tested (28). However, GS-6620, which was well tolerated in animals and in phase 1 clinical studies (29), had cytostatic effects in MRC5, MT-4, and bone marrow cells. Furthermore, the nucleosides released by valopicitabine and balapiravir, the nucleoside prodrugs whose clinical development was stopped due to toxicity, did not show marked cytotoxicity in the different cell types tested. In particular, 4′-azidoC showed no cytotoxicity in hematologic cells to correlate with the clinical findings reported with balapiravir. 2′CMeC was toxic to the MT-4 lymphocyte-derived cell line, with a CC_50_ of 19 μM, but there was little or no cytotoxicity in other cell types. BMS-986094 showed marked cytotoxicity in all human cell lines and primary cells tested. However, an alternate prodrug of 2′CMeG, IDX184, showed minimal toxicity. The lack of effect of IDX184 was likely due to inefficient activation in the cells studied (Table 3). Based on the clinical observation of cardiomyopathy in patients treated with BMS-986094, its cytotoxicity was also assessed in primary rat cardiomyocytes (30). After 5-day treatments with BMS-986094, cardiomyocytes showed the most profound sensitivity to cytotoxicity among the primary cells tested, with a CC_50_ of 0.68 ± 0.32 μM (mean ± standard deviation), almost equivalent to that of puromycin (CC_50_ of 0.33 ± 0.05 μM).

**TABLE 1:** Cytotoxicity in human cell lines after 5-day treatment

Base	Compound	Mean CC_50_ ± SD (μM) in:				
		Hepatic cells		Prostate cells (PC-3)	Fibroblasts (MRC5)	T-cells (MT-4)
		Huh7	HepG2 (galactose)			
Uridine	Sofosbuvir	66 ± 17	>89	>100	>89	>100
Guanosine	IDX184	>89	>89	77 ± 14	>89	66 ± 18
	BMS-986094	0.5 ± 0.2	1.9 ± 1.0	1.0 ± 0.2	1.1 ± 0.3	1.1 ± 0.5
	PSI-938	>89	>89	>100	>89	>100
Cytidine	PSI-6130	>89	>89	>100	>89	>100
	4′-AzidoC	>89	>89	30 ± 6	>89	>100
	2′CMeC	>89	>89	49 ± 18	69 ± 1	19 ± 6
Adenosine	GS-6620^*a*^	67 ± 13	66 ± 13	40 ± 0.7	8.9 ± 4.1^*b*^	7.8 ± 4.3^*b*^
	ddC^*c*^	>89	>89	0.80 ± 0.21	>89	>89
	Chloramphenicol^*c*^	>89	>89	16 ± 3	>89	>89
	Αlpha-amanitin^*c*^	0.45 ± 0.16	0.81 ± 0.35	0.17 ± 0.06	0.34 ± 0.07	0.89 ± 0.10
	Puromycin^*c*^	0.52 ± 0.19	0.65 ± 0.18	0.22 ± 0.07	0.24 ± 0.05	0.16 ± 0.06

a The cytotoxicity of GS-6620 in some cell types was reported previously (13).

b Complex, multiphasic dose response was observed, with an initial drop in viability of 50% followed by a rebound to over 50% at higher concentrations. Flow cytometry studies suggested that the effect of the compound on these cells was cytostatic instead of cytotoxic.

c Control treatment.

**TABLE 2:** Cytotoxicity in human primary cells^*a*^

Base	Compound	Mean CC_50_ ± SD (μM) in:				
		Hepatic cells	PBMCs		Bone marrow cells	
			Quiescent	Stimulated	Erythroid	Myeloid
Uridine	Sofosbuvir	>100	>100	>100	>100^*b*^	>100^*b*^
Guanosine	IDX184	>100	>100	>100	100 ± 10	>100
	BMS-986094	6.7 ± 3.2	11 ± 4	7.8 ± 2.0	4.5 ± 1.5	2.6 ± 0.5
	PSI-938	>100	>100	>100	>100	>100
Cytidine	PSI-6130	>100	>100	100	ND^*c*^	ND
	4′-AzidoC	>100	>100	>100	>100	108 ± 12
	2′CMeC	>100	>100	64 ± 18	99 ± 18	76 ± 16
Adenosine	GS-6620^*d*^	>100	>100	>100	15 ± 7	3.1 ± 2.6
	ddC^*e*^	>100	>100	>100	1.9 ± 0.3	9.7 ± 2.0
	Chloramphenicol^*e*^	>100	>100	>100	82 ± 29	>100
	Alpha-amanitin^*e*^	0.12 ± 0.01	0.33 ± 0.11	0.12 ± 0.03	0.57 ± 0.24	0.51 ± 0.07
	Puromycin^*e*^	1.9 ± 0.8	4.5 ± 1.9	1.0 ± 0.2	0.36 ± 0.06	0.27 ± 0.04
	5-FU^*e*^	>100	>100	>100	3.7 ± 0.5	3.4 ± 0.8

a Hepatocytes and PBMCs were treated for 5 days, while bone marrow-derived cells were treated for 14 days.

b Data are from reference 28.

c ND, not determined.

d Data are from reference 13.

e Control treatment.

**TABLE 3:** HCV NI intracellular activation in PC-3 cells

Base	HCV NI	Mean TP concn (pmol/million) ± SD^*a*^
Uridine	Sofobuvir	84.7 ± 0.4
Guanosine	IDX184	28 ± 2
	BMS-986094	1,050 ± 30
	PSI-938	0.86 ± 0.32
Cytidine	PSI-6130	71 ± 9
	4′-AzidoC	10 ± 1
	2′CMeC	83 ± 7
Adenosine	GS-6620	BLQ

a The amount of nucleoside triphosphate was determined from three independent LC-MS/MS analyses. The reported TP levels are the maximal concentration observed after 48 h during a 5-day incubation. BLQ, below the limit of quantification.

### Measurement of active triphosphate metabolite in PC-3 cells.

Prodrug activation and nucleotide phosphorylation can be highly cell type dependent and can lead to different degrees of cytotoxicity. However, it is impractical to measure this process in multiple cell lines. In this study, we chose to measure the triphosphate concentration after 48-h incubations of compounds with PC-3 cells. As shown by the results in Table 3, sofosbuvir and PSI-6130 efficiently formed TP, illustrating that the lack of toxicity (CC_50_ <100 μM) (Table 1) was not due to poor intracellular activation. In contrast, the TP level of IDX184 was >37-fold less than that of BMS-986094, which correlated well with the >47-fold difference in the CC_50_ values in PC-3 cells.

### Inhibition of human DNA and RNA polymerases and incorporation by PolRMT.

Cell-based assays for general cytotoxicity failed to reliably identify NI associated with clinical toxicity. Assessing the interactions of the active triphosphate metabolites of the test compounds with the potential molecular targets of toxicity may be a more sensitive way of assessing the potential for off-target effects. As summarized in Table 4, none of the active triphosphates inhibited human DNA polymerases alpha, beta, and gamma at the highest concentration tested (100 μM), nor did they affect PolII-catalyzed RNA synthesis (50% inhibitory concentration [IC_50_] of >200 μM).

**TABLE 4:** Inhibition of human DNA and RNA polymerases and substrate utilization of 5′-triphosphate active metabolites of HCV NI

Base	Triphosphate form of the compound	Mean IC_50_ ± SD (μM) for^*a*^:					Substrate incorporation (% of NTP [mean ± SD]) of PolRMT
		DNA Pol α	DNA Pol β	DNA Pol γ	RNA PolII	PolRMT	
Uridine	Sofosbuvir	>200	>200	>200	>200	>500	0.45 ± 0.40
Guanosine	IDX184 BMS-986094	>200	>200	>200	>200	52 ± 7	92 ± 33
	PSI-938	>200	>200	>200	>200	>500	1.6 ± 0.2
Cytidine	PSI-6130/RG7128	>200	>200	>200	>200	>500	0.81 ± 0.13
	4′-azidoC	>200	>200	>200	>200	3.8 ± 2.0	103 ± 9
	2′CMeC	>200	>200	>200	>200	230 ± 90	84 ± 1
Adenosine	GS-6620	>200	>200	>200	>200	>500	0.03 ± 0.02
	Aphidicolin^*b*^	4.7 ± 3.3					
	3′dTTP^*b*^		1.9 ± 0.8				
	3′dTTP^*b*^			1.2 ± 0.6			
	Alpha-amanitin^*b*^				0.0035 ± 0.0015		
	3′deoxyGTP^*b*^					4.2 ± 1.4	

a Pol α, polymerase alpha; Pol β, polymerase beta; Pol γ, polymerase gamma; PolII, polymerase II; PolRMT, mitochondrial RNA polymerase.

b Specific positive control.

In contrast to DNA polymerases and RNA PolII, PolRMT was shown to interact with the active forms of many of the NI. The triphosphate formed by 4′-azidoC was a potent inhibitor of PolRMT-catalyzed RNA synthesis, with an IC_50_ of 3.8 μM. The active triphosphates of BMS-986094 and IDX184 and 2′CMeC were also inhibitors, with IC_50_s of 52 and 230 μM, respectively. In addition, we directly measured the incorporation of the triphosphates in biochemical assays at a fixed saturating concentration (500 μM) and compared the relative rates of incorporation to those of the corresponding natural ribonucleoside triphosphates (rNTPs). As shown by the results in Table 4, triphosphates formed by BMS-986084 and IDX184, 4′-azidoC, and 2′CMeC served as excellent substrates and were incorporated by PolRMT at rates similar to those of their corresponding natural rNTPs. In contrast, the active forms of sofosbuvir, PSI-938, mericitabine, and GS-6620 were all exceedingly poor substrates for PolRMT (rates of <1% of their respective rNTPs).

### Structural modeling of PolRMT.

**FIG 2:**
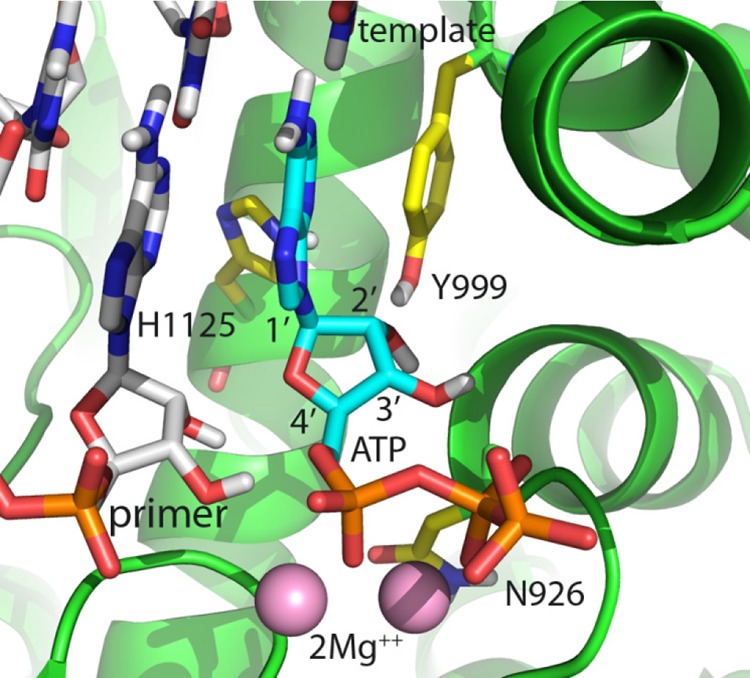
Homology model of ATP incorporation into human PolMRT based on a crystal structure of T7 RNA polymerase (PDB ID 1S76). The active site is well conserved, with only N926 (shown) and Q992 (not shown, but positioned above the substrate/template bases) being different. A 1′CN substitution shifts H1125 toward 2′. A 2′CMe substitution shifts Y999 toward 1′. When combined, these two substitutions are then in conflict and the analog is a poor substrate.

We constructed a homology model of PolRMT based on a ternary structure of T7 RNA polymerase (26). An examination of various NI presented here provided some insight into their relative activities (Fig. 2). Monosubstituted ribose analogs could be accommodated by the PolRMT active site to various degrees. The 4′-azido substitution was the most easily accommodated by the enzyme, with essentially no perturbation of the active site required for the inhibitor to bind. The 2′CMe substitution has a slight van der Waals clash with Tyr999, leading to a small shift in this residue toward 1′. The movement of this residue was more pronounced with the 2′F,2′CMe substitutions, likely due to the loss of hydrogen bonding capacity with the fluorine. Similarly, a 1′CN substituent leads to a slight van der Waals clash with His1125, thus leading to a small shift in this residue toward 2′. Consistent with the biochemical result of exceedingly poor incorporation for the active metabolite of GS-6620, the opposing movements of Tyr999 and His1125 would not be tolerated in the presence of both 1′ and 2′ substitutions.

### Effects on mitochondrial protein synthesis.

In order to determine whether their interaction with PolRMT had any consequences for protein expression, we measured the effects of HCV NI on the levels of mitochondrial (transcribed by PolRMT) and nuclear (transcribed by PolII) DNA-encoded proteins in cultured cells. Mitochondrial toxicity studies have typically been conducted in the liver cell line HepG2. However, based on the greater sensitivity of PC-3 cells to general cytotoxicity to HCV NI, studies using the mitochondrial toxin dideoxycytosine (ddC) were done in these cells to determine whether they would provide a more sensitive model for mitochondrial toxicity. PC-3 cells were >30-fold more sensitive than HepG2 cells to mitochondrial DNA depletion after a 10-day treatment (see Fig. S1A in the supplemental material), and they were >500-fold more sensitive to reductions in COX-1 protein expression after a 5-day treatment (see Fig. S1B and C). Based on these results, PC-3 cells were chosen for subsequent cellular studies.

**FIG 3:**
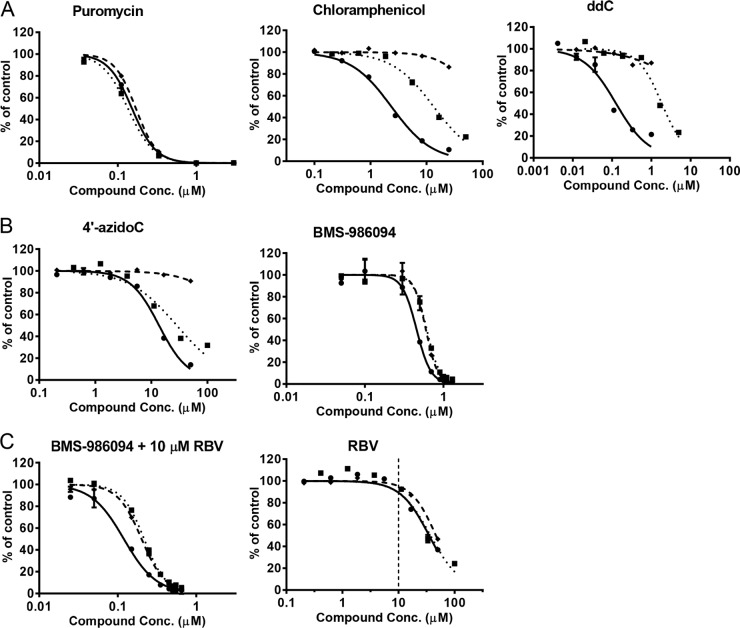
Effects of compounds on mitochondrial protein synthesis in PC-3 cells after 5-day treatments. The levels of mitochondrial protein synthesis, represented by COX-1 protein (•), nuclear protein synthesis, represented by SDH-A protein (◆), and ATP (■) were curve fitted with solid lines, dashed lines, and dotted lines, respectively. (A) Puromycin showed nonselective inhibitive effects on the COX-1, SDH-A, and ATP levels, while chloramphenicol and ddC specifically inhibited COX-1 synthesis. (B) 4′-AzidoC and BMS-986094 showed selective inhibition of COX-1 synthesis. (C) RBV at a concentration not resulting in any observed toxicity (10 μM) potentiated the mitochondrial and general cellular toxicity associated with BMS-986094. In contrast, at 10 μM, RBV showed no appreciable effect on mitochondrial or nuclear protein synthesis or ATP level.

The effects on cellular protein production of model inhibitors of different cellular processes, including mitochondrial replication (ddC), mitochondrial translation (chloramphenicol), and general cellular translation (puromycin), were characterized (Fig. 3A). Consistent with their targets in mitochondria, ddC and chloramphenicol caused selective depletion of mitochondrial-DNA-encoded COX-1 prior to any effect on nuclear-DNA-encoded SDH-A or ATP. In contrast, puromycin had no selective effect with COX-1, SDH-A, and ATP, as shown by superimposable dose-response curves. The nonnucleotide RNA PolII inhibitor alpha-amanitin had a profile similar to that of puromycin (data not shown).

As summarized in Table 5, many of the HCV NI tested in this system showed little or no cytotoxicity and no evidence for a selective effect on mitochondrial protein synthesis. Similar to the control mitochondrial toxins ddC and chloramphenicol, 4′-azidoC (nucleoside of balapiravir) showed selective inhibition of mitochondrial protein synthesis, with a CC_50_ of 11 μM, while no change in nuclear protein translation was observed at up to 50 μM (Table 5; Fig. 3B). BMS-986094 showed a mixed effect, somewhere between those of the selective mitochondrial toxins ddC and chloramphenicol and the general cellular cytotoxicity of puromycin (Fig. 3B). The slightly enhanced sensitivity to BMS-986094 of COX-1 relative to that of SDH-A was reproducible in three independent studies done in duplicate (*F* test, *P* < 0.0001). This effect on mitochondrial protein production is consistent with the reported effect of another 2′CMe-monosubstituted analog, 2′CMe adenosine, which showed selective depletion of mitochondrial RNAs in a prior study (12).

**TABLE 5:** Effects of HCV NI on mitochondrial protein production, respiration, cellular protein production, and ATP levels in PC-3 cells

Base	HCV NI	Mean CC_50_ ± SD (μM) causing:			
		Mitochondrial toxicity		General cellular toxicity	
		Protein synthesis^*a*^	Respiration^*b*^	Protein synthesis^*a*^	ATP level^*a*^
Uridine	Sofosbuvir	>100	ND^*c*^	>100	>100
Guanosine	IDX184	96 ± 3	ND	>100	77 ± 14
	BMS-986094	0.68 ± 0.10	0.48 ± 0.07	0.84 ± 0.26	1.0 ± 0.2
	PSI-938	>100	ND	>100	>100
Cytidine	PSI-6130	70 ± 14	ND	>100	>100
	4′-AzidoC	11 ± 3	4.5 ± 0.3	>50	30 ± 6
	2′CMeC	50 ± 25	100	68 ± 26	49 ± 18
Adenosine	GS-6620	31 ± 5	ND	45 ± 2	40 ± 0.7
	ddC^*d*^	0.19 ± 0.07	0.21 ± 0.03	>1	0.80 ± 0.21
	Chloramphenicol^*d*^	2.6 ± 0.6	4.5 ± 1.5	>50	16 ± 3
	Puromycin^*d*^	0.12 ± 0.01	0.49 ± 0.11	0.15 ± 0.01	0.22 ± 0.07

a Cells were treated with compounds for 5 days.

b Cells were treated with compounds for 3 days.

c ND, not determined.

d Control treatment.

To further assess the mechanism of BMS-986094 toxicity, this compound was studied in combination with RBV. RBV is an inhibitor of IMP dehydrogenase (IMPDH) and reduces endogenous GTP pools that compete with the pharmacologically active triphosphate formed by BMS-986094. While RBV itself had low cytotoxicity and no selective effect on mitochondria, its coincubation with BMS-986094 potentiated the mitochondrial and cellular toxicity of BMS-986084 by 3- to 4-fold (Fig. 3C).

### Inhibition of mitochondrial respiration.

**FIG 4:**
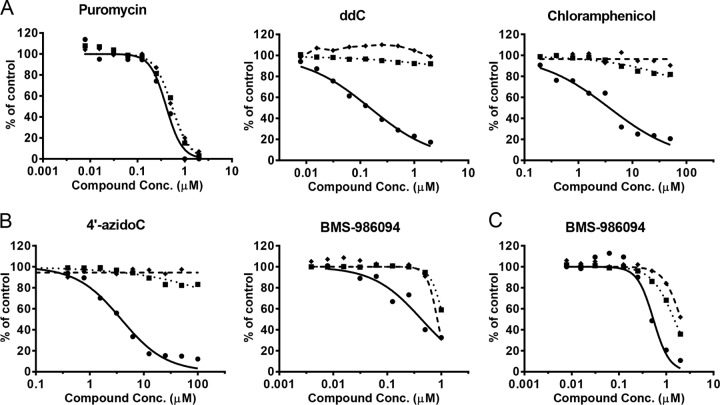
Effects of compounds on mitochondrial spare respiratory capacity in PC-3 cells (A and B) and primary rat cardiomyocytes (C) after 3-day treatments. The relative levels of spare respiratory capacity (•), DNA (◆), and ATP (■) were curve fitted with solid lines, dashed lines, and dotted lines, respectively. The spare respiratory capacity was normalized by cell numbers. (A) Puromycin showed nonselective inhibition of the spare respiratory capacity, DNA, and ATP levels, while ddC and chloramphenicol specifically inhibited mitochondrial spare respiratory capacity but showed minimal effects on cellular DNA and ATP levels. (B) 4′-AzidoC and BMS-986094 showed selective inhibition of mitochondrial respiration in PC-3 cells. (C) BMS-986094 showed selective inhibition of mitochondrial respiration in primary rat cardiomyocytes.

In order to assess whether there was any functional consequence of the inhibition of mitochondrial protein production, mitochondrial spare respiratory capacity was assessed after 3-day treatments with the NI (Fig. 4; Table 5). A 3-day instead of 5-day time point was chosen due to the high sensitivity of the assay. The control inhibitors ddC, chloramphenicol, and puromycin showed results consistent with their effects on mitochondrial protein synthesis discussed above. For example, ddC and chloramphenicol selectively reduced respiration at concentrations that did not affect total cellular DNA content or ATP (Fig. 4A). Correlating with the biochemical and protein expression data, 4′-azidoC showed selective effects on respiration similar to those observed with the positive controls (Fig. 4B). Consistent with the results from mitochondrial protein synthesis assays, BMS-986094 showed a primary effect on mitochondrial respiration followed by general cytotoxicity in both PC-3 cells (CC_50_ of 0.48 ± 0.07 μM) (Fig. 4B) and primary rat cardiomyocytes (CC_50_ of 0.43 ± 0.14 μM) (Fig. 4C). All other HCV NI, including those showing some cytotoxicity in PC-3 cells, did not have a selective effect on mitochondrial respiration.

### Inhibition of RNA polymerases by 2′CMeNTP analogs.

In the above-described studies, BMS-986094 showed a profile that was intermediate between the profiles of selective mitochondrial toxins like ddC, chloramphenicol, and 4′-azidoC and those of generally cytotoxic agents, exemplified by puromycin. Furthermore, the effects of BMS-986094 were increased by depleting the levels of GTP with ribavirin, suggesting that the effects are caused by competition at the nucleotide level. Combined, these results suggest that BMS-986094 and other 2′CMe monosubstituted NI may have an off-target polymerase in addition to PolRMT. Therefore, 2′CMe monosubstituted NTPs of the four natural ribonucleotide bases (2′CMeGTP, 2′CMeATP, 2′CMeCTP, and 2′CMeUTP) were tested for their inhibition of RNA PolI, PolII, PolIII, and PolRMT. As summarized in Table 6, all four 2′CMeNTPs showed inhibition of PolI but not of PolII or PolIII.

**TABLE 6:** Inhibition of RNA polymerases by 2′CMe-modified nucleotide analogs

Nucleotide analog	Mean IC_50_ ± SD (μM) for:			
	RNA PolI	RNA PolII	RNA PolIII	PolRMT
2′CMeGTP	300 ± 60	>500	>500	52 ± 7
2′CMeATP	190 ± 40	>500	>500	53 ± 14
2′CMeCTP	260 ± 40	>500	>500	230 ± 90
2′CMeUTP	690 ± 230	>500	>500	240 ± 30
3′-deoxyGTP	25 ± 2	>500	104 ± 20	4.2 ± 1.4

### Detection of RNA transcripts in PC-3 cells.

**FIG 5:**
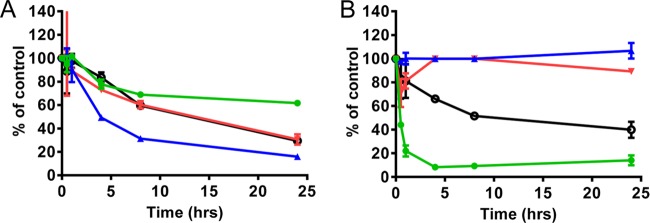
Effects of BMS-986094 on RNA transcript levels in PC-3 cells during a 1-day treatment. The relative levels of transcripts of preribosome (PolI), myc (PolII), mitochondrial ATP6 (PolRMT), and total RNA are shown in green (•), blue (▲), red (▼), and black (⭘). Cells were treated with BMS-986094 (100 μM) (left) or the positive control RNA Pol1 inhibitor CX-5461 (5 μM) (right), and transcripts in purified total nuclear RNA were quantified by RT-qPCR.

The BMS-986094-induced inhibition of the above-mentioned RNA polymerases was further tested in cell culture, where representative RNA transcription products from PolI (preribosome), PolII (myc), and PolRMT (ATP6) in PC-3 cells were measured using RT-PCR during a 1-day treatment. As shown by the results in Fig. 5, 100 μM BMS-986094 decreased the relative levels of multiple RNA transcripts, including preribosomal, myc, and mitochondrial ATP6, and total RNA, while the different RNA levels at 24 h may reflect the different degrees of stability of these RNA products. In contrast, at 5 μM, the positive-control RNA PolI inhibitor CX-5461 selectively decreased pre-rRNA but had no effect on the levels of myc and mitochondrial ATP6 RNAs.

## DISCUSSION

Nucleotide analogs have played key roles as antiviral agents for herpes simplex virus (HSV), human immunodeficiency virus (HIV), and hepatitis B virus (HBV) (31). However, the early generations of NI targeting these viruses were plagued by toxicity (32). The toxicity potential of early 2′-deoxyribonucleotide analogs was perhaps best exemplified by the delayed liver failure observed during a clinical trial of fialuridine (FIAU) for HBV (33). Preclinical studies had not predicated this toxicity. Elucidation of the mechanism of FIAU toxicity, mitochondrial dysfunction caused by incorporation by the mitochondrial DNA polymerase gamma, enabled the development of cell-based and biochemical assays that serve as effective counterscreens to allow the development of more selective NI (34–37). Different from the NI targeting DNA viruses, all HCV NI are ribonucleotide analogs and, therefore, are more likely to target host RNA polymerases than DNA polymerases. An analogous understanding of key modes of toxicity for ribonucleoside analogs would serve to improve the chances of successful clinical development for indications like HCV.

In contrast to our understanding of the inhibition of DNA polymerases by dNTP analogs, little is known about the inhibition of RNA polymerases by rNTP analogs. In the few published studies where inhibition of RNA polymerases has been assessed, only RNA PolII and RNA poly(A) polymerase were studied and no inhibition of any appreciable level was noted for select analogs (28, 38). It has recently been reported that PolRMT can incorporate many modified rNTP analogs, including triphosphates formed by 4′- and 2′-modified NI explored as anti-HCV agents (12). This effect on PolRMT may be a valuable clue to the mechanism for the clinical toxicity observed with some ribonucleotide analogs.

In an effort to identify the best predictive toxicity parameters *in vitro*, we conducted multiple cell- and enzyme-based studies on clinically tested HCV NI with publically disclosed chemical structures. Similar to the observation with 2′-deoxynucleotide analogs in the past, cytotoxicity in human cell lines and primary cells was found to be an unreliable and insensitive predictor of toxicity potential. Two HCV NI that have had their clinical development terminated, 2′CMeC and 4′-azidoC, only showed inconsistent cytotoxicity against the panel of cells studied here. Of note, 4′-azidoC, the nucleoside released by the prodrug balapiravir, whose clinical trial was stopped due to clinical hematologic toxicity, did not show effects on lymphoid cell lines or primary bone marrow-derived erythroid and myeloid cells. Conversely, GS-6620 showed cytostatic effects on fibroblast- and lymphocyte-derived cell lines and cultured primary bone marrow cells but did not show effects on PBMCs *in vitro* or any corresponding effects during chronic toxicology studies of up to 39 weeks in rats and dogs. In general, PC-3 cells were found to be the most sensitive to HCV NI toxicity among the cell types tested. For example, PC-3 was the only cell line to show any cytotoxicity from the selective mitochondrial toxins ddC, chloramphenicol, and 4′-azidoC. Liver-derived HepG2 cells have historically been used as a standard for cytotoxicity evaluation; however, our study shows that they are less sensitive to the known mitochondrial toxin ddC and certain HCV NI. Presently, we do not have an explanation for this observation, since the two cell lines are very similar in the following areas: (i) the glucose concentration in the culture medium is 5 to 7 mM, (ii) the two cell lines have similar doubling times, (iii) both cell lines showed similar oxygen consumption rates and rates of glycolysis, and (iv) both cell types have been found to efficiently activate nucleoside analogs (data not shown).

The inability of *in vitro* toxicity assays to reliably identify NI associated with clinical adverse events may be due to many reasons, including (i) insufficient TP formation caused by cell type-dependent prodrug activation and nucleotide phosphorylation (Table 3), (ii) altered signaling cascades and cell death regulation in immortal cell lines, (iii) cell-specific sensitivity to mitochondrial insult, and (iv) altered metabolic pathways under cell culture conditions, including the presence of glucose-rich cell culture media (39). By avoiding these limitations, biochemical assays assessing the molecular determinant(s) of toxicity would serve as the most reliable indicator of toxicity potential if target(s) could be identified. Given inhibition of the viral polymerase as the mechanism of action for NI, we studied a number of host polymerases for interactions with HCV NI. In these studies, none of the three human DNA polymerases (alpha, beta, and gamma) or PolII was inhibited by the TP formed by the HCV NI tested. In contrast, PolRMT incorporated all of the 2′CMeNTP analogs and 4′-azido CTP efficiently, making PolRMT a candidate for the molecular target resulting in the observation of adverse events with HCV NI.

Assessing direct effects on mitochondrial gene products and function allowed the confirmation of the relevance of the PolRMT biochemical results. In particular, the consequences for mitochondrial function from the interactions with PolRMT were established for the active metabolites of 4′-azidoC and BMS-986094. Illustrating the importance of intracellular activation for the mitochondrial toxicity observed in cell culture, IDX184 and BMS-986094 are both prodrugs of 2′CMeGTP, and yet there was a difference of 2 orders of magnitude in their effects on mitochondrial COX-1 expression, reflecting the different levels of activation noted in PC-3 cells (Tables 3 and 5). Consistent with the different toxicities of these prodrugs, BMS-986094 has been reported to be 30- to 60-fold more potent than IDX184 against the HCV replicon (40, 41).

In the clinic, some HCV NI have demonstrated adverse effects on distinct organ systems, including gastrointestinal, hepatic, cardiac, and hematological tissues. While these diverse toxic effects may be related to different underlying mechanisms of insult, it is also possible that one or a few off-target activities exist and that the sensitive organ is based on drug distribution. For example, although both chloramphenicol and ddC are mitochondrial toxins, they display distinct clinical toxicities, with chloramphenicol causing anemia and ddC causing peripheral neuropathy. Likewise, the 2′-deoxynucleoside analogs used for HBV and HIV have been associated with anemia, neuropathy, myopathy, cardiomyopathy, lipid abnormalities, liver toxicity, renal toxicity and peripheral neuropathy, all apparently caused by inhibition of a single target, the mitochondrial DNA polymerase gamma (42). Like all methods designed to assess a specific mechanism of toxicity, our studies have limitations. For example, despite having tested it in a comprehensive battery of biochemical and cell-based assays, we were unable to elucidate a mechanism for the clinically observed adverse hepatic effects observed with PSI-938.

When assessing the potential toxicity of NIs, comprehensive testing across multiple platforms and cell lines is critical. Based on a review of available data, Ahmad et al. recently concluded that direct mitochondrial toxicity is unlikely to be the mechanism of the cardiac effects of BMS-986094 (30). This conclusion is at odds with the findings in the current study and an earlier report showing BMS-986094-induced lactic acid increases in HepG2 cells (43). Our study showed efficient incorporation of the triphosphate of BMS-986094 by PolRMT, primary effects on mitochondrial protein production, and an initial decrease in respiration in PC-3 cells and rat cardiomyocytes prior to decreases in cell viability. Furthermore, the marked shift in the mitochondrial effects caused by ribavirin, an inhibitor of IMP dehydrogenase, and the subsequent reduction of competing endogenous GTP pools further support the role of triphosphate incorporation by a host polymerase. Given the high requirements for mitochondrion-derived energy in cardiac tissue, it is hard to dismiss these findings as a causative factor. Of note, other mitochondrial toxins, including ddC and chloramphenicol, have been reported to cause cardiomyopathy (44–46). While the primary effect of BMS-986094 was on mitochondrial function, the profile of this compound is intermediate between those of highly selective mitochondrial toxins (e.g., ddC, 4′-azidoC, and chloramphenicol) and generally cytotoxic agents (e.g., alpha-amanitin and puromycin), suggesting a secondary target for toxicity. Further experiments suggested that the general effects of BMS-986094 may be mediated by inhibition of other RNA polymerases, based on the inhibition of RNA PolI by 2′CMe monosubstituted analogs in biochemical assays (Table 6) and the depletion of RNA transcripts generated by a number of RNA polymerases in cellular studies (Fig. 5).

In conclusion, a comprehensive study of the *in vitro* toxicity potential of HCV NI has identified PolRMT as a potential off-target mediator of tissue-specific adverse events observed clinically. Analogs that have been associated with adverse events, including valopicitabine, balapiravir, and BMS-986094, were observed to interfere with PolRMT in cell-based and biochemical assays. In contrast, mericitabine and sofosbuvir did not interact with PolRMT or cause mitochondrial toxicity, consistent with their progression into advanced clinical development. We propose that a screening paradigm for HCV NI and, more broadly, other drug classes should include specific monitoring of mitochondrial transcription and respiration in PC-3 cells and biochemical assays studying the interactions with PolRMT. A more comprehensive assessment of mitochondrial toxicity would reduce the observation of previously unappreciated toxicity during clinical trials and, thus, aid in the successful development of new therapies.

## Supplementary Material

Supplemental material
