# Supplementary material for: Role of Mitochondrial RNA Polymerase in the Toxicity of Nucleotide Inhibitors of Hepatitis C Virus
Source: Antimicrob Agents Chemother. 2016 Jan 29;60(2):806–17. doi: 10.1128/AAC.01922-15 (PMC4750701; doi:10.1128/AAC.01922-15)
Supplement: Supplemental material [file supp_60_2_806__index.html]

Role of Mitochondrial RNA Polymerase in the Toxicity of Nucleotide Inhibitors of Hepatitis C Virus — Supplemental material 

# Role of Mitochondrial RNA Polymerase in the Toxicity of Nucleotide Inhibitors of Hepatitis C Virus

## Supplemental material

- Supplemental file 1 -

  Supplemental Materials and Methods, Fig. S1, Table S1

  PDF, 57K
