# Supplementary material for: Role of Mitochondrial RNA Polymerase in the Toxicity of Nucleotide Inhibitors of Hepatitis C Virus
Source: Antimicrob Agents Chemother. 2016 Jan 29;60(2):806–17. doi: 10.1128/AAC.01922-15 (PMC4750701; doi:10.1128/AAC.01922-15)
Supplement: Supplemental material [file AAC.01922-15_zac002164804so1.pdf]

**Supplementary Data to:**

# Role of the Mitochondrial RNA Polymerase in the Toxicity of Nucleotide Inhibitors of the Hepatitis C Virus

Joy Y. Feng, Yili Xu, Ona Barauskas, Jason K. Perry, Shekeba Ahmadyar, George Stepan, Helen Yu, Darius Babusis, Yeojin Park, Krista McCutcheon, Michel Perron, Brian E. Schultz, Roman Sakowicz, and Adrian S. Ray

**Supplementary Materials and Methods**

*Cell Culture and Cytotoxicity Studies*

The Huh-7 cells were maintained in growth medium containing Dulbecco's Modified Eagle Medium (DMEM, Cellgro) supplemented with 1% Nonessential Amino Acids (NEAA, Cellgro, Manassas, VA), 10% fetal bovine serum (FBS, HyClone, Logan, UT) and 1% Penicillin-streptomycin-glutamate (PSG, Invitrogen, Carlsbad, CA). The cells were passaged twice weekly and maintained at 90% confluence densities. The glucose concentration in DMEM medium was 25 mM.

The HepG2 cells were maintained in Eagle's Minimum Essential Medium (EMEM) from ATCC (Manassas, VA) supplemented with 10% FBS, 100 units/mL penicillin and 100 µg/mL streptomycin (P/S) (Gibco). The cells were passaged twice weekly and maintained at 90% confluence. The glucose concentration in EMEM medium was 5.5 mM.

The PC-3 cells were maintained in Kaighn's F12 medium (Gibco, Carlsbad, CA) supplemented with 10% FBS (HyClone, Logan, UT), 100 units/mL penicillin and 100 µg/mL streptomycin (P/S) (Gibco). The cells were passaged twice weekly and maintained at 90% confluence. Cells between passages 4 and 15 were used in the assays. The glucose concentration in Kaighn's F12 medium was 7 mM.

The fibroblast cell line MRC-5 cell line derived from normal lung tissue, was purchased from ATCC (Manassas, VA) and cultured in Minimum Essential Medium (MEM) (Cellgro, Manassas, VA) supplemented with 10% FBS (HyClone, Logan, UT) and 1% PSG (Invitrogen). The cells were passaged twice weekly and maintained at 90% confluence densities. The glucose concentration in MEM medium was 5.5 mM.

MT-4 cells are HTLV-1 transformed human T lymphoblastoid cells obtained from the NIH AIDS Research and Reference Reagent Program (Germantown, MD), and maintained in RPMI-1640 medium with GlutaMAXTM (Gibco, Carlsbad, CA) supplemented with 10% FBS (HyClone, Logan, UT) and 100 units/mL penicillin and 100 µg/mL streptomycin (P/S, Gibco, Carlsbad, CA). MT-4 cells were passaged twice a week and maintained at densities below $0.6 \times 10^6$ cells/mL. The glucose concentration in RPMI-1640 medium was 11 mM.

Human primary hepatocytes were obtained from the following two vendors along with vendor-specific culture medium and supplements: (1) Bioreclamation IVT (Westbury, NY). The cells were supplied in a 96-well format with a Matrigel overlay, maintained in InVitroGro CP or HP medium and antibiotic Mix Torpedo. The cells were seeded at a density of $3.75 \text{ x } 10^4$ cells per well. The glucose concentration was 5.5 mM; (2) Invitrogen, Carlsbad, CA. The cells were supplied in a 96-well format with a Geltrex overlay, maintained in William’s Medium E and Cell Maintenance Supplement provided by the vendor. The cells were seeded at a density of $3.75 \times 10^4$ cells per well. The glucose concentration in the William’s Medium E was 11 mM.

Human peripheral blood mononuclear cells (PBMCs) were isolated from human buffy coats obtained from normal healthy volunteers (Stanford Medical School Blood Center, Palo Alto, CA). Total PBMCs were isolated from buffy coats using standard Ficoll separation and sterile cellular isolation techniques performed in a bio-safety cabinet (1). Except where noted, the procedure was carried out at room temperature. The final PBMCs pellet was resuspended in RPMI 1640 with Glutamax™ (Gibco, Carlsbad, CA) supplemented with 10% FBS (Hyclone, Logan, UT), penicillin 100 units/mL and streptomycin 100 µg/mL (Gibco). For quiescent

PBMCs cultures, total cells were seeded in 96-well plates at a density of 1 x $10^6$ cells per well, followed by immediate drug treatment. Stimulated PBMCs cultures were set up by seeding the freshly isolated PBMCs in T-175$cm^2$ tissue culture flasks in 100 mL of RPMI containing the same components as the ones used for the quiescent PBMCs but with the addition of 10 units per mL of recombinant human interleukin-2 (hIL-2, Roche, Indianapolis, IN), and 1 µg per mL phytohemagglutinin-P (PHA-P, Sigma-Aldrich, St. Louis, MO) at density of $2 \times 10^6$ cells per mL for 48 hours at 37°C in a 5% $CO_2$ incubator with 90% humidity, prior to drug treatment. The glucose concentration in RPMI-1640 medium was 11 mM.

Normal human primary bone marrow (BM) light density cells from three different lots obtained from AllCells (Emeryville, CA) or Lonza (Walkersville, MD) were stored at -152ºC until required for the assay. On the day of the experiment, the cells were thawed rapidly at 37ºC, the contents of the vial were diluted in 10 mL of Iscove's Modified Dulbecco's Medium containing 2% fetal bovine serum (IMDM + 2% FBS) and washed by centrifugation (1200 r.p.m. for 10 minutes at room temperature). The supernatant was discarded and the cell pellet was resuspended in a known volume of IMDM + 2% FBS. A nucleated cell count (3% glacial acetic acid) and viability assessment (trypan blue exclusion test) were performed. The glucose concentration in IMDM medium was 17 mM.

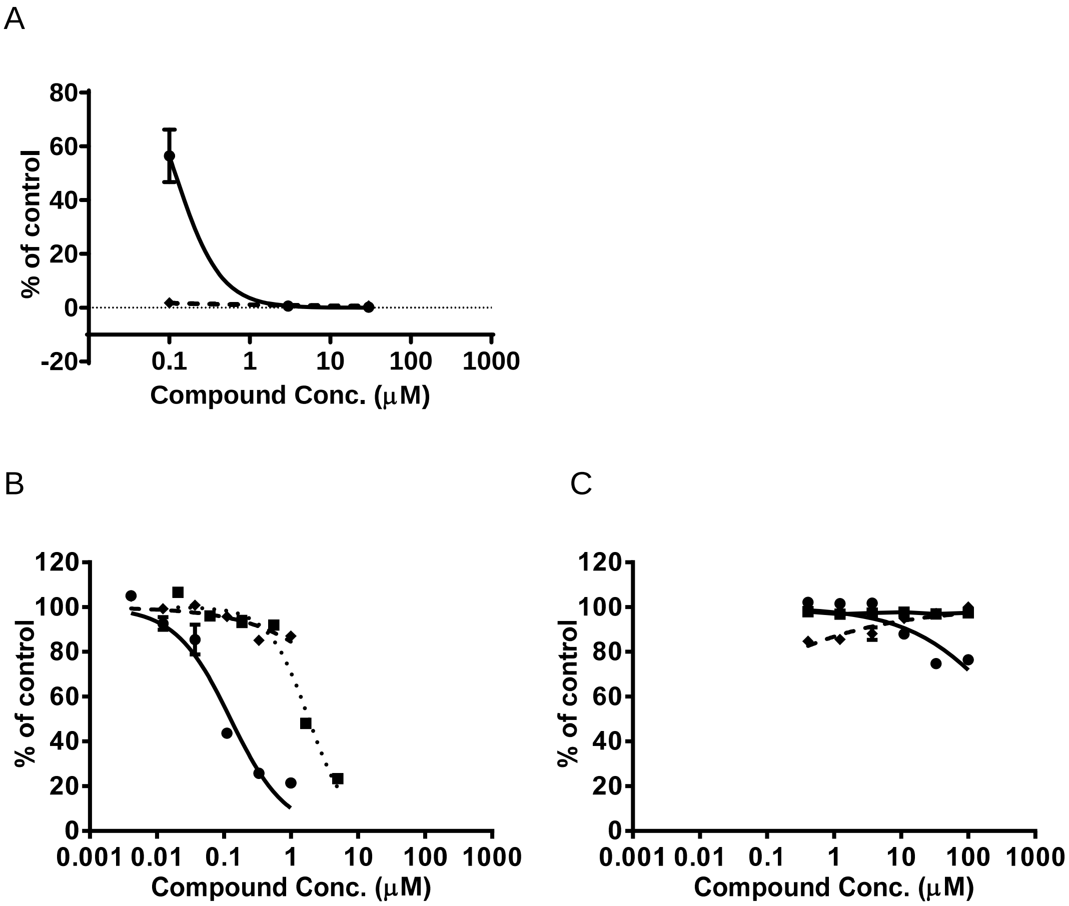


**Supplementary Fig. S1: PC-3 cells are more sensitive to ddC-induced mitochondria toxicity than HepG2 cells.** (A) Levels of mitochondrial DNA in PC-3 cells (shown in ♦ and dashed line) and HepG2 cells (shown in • and solid line) after 10-day treatment; Levels of mitochondrial protein synthesis in PC-3 cells (B) and HepG2 cells (C) represented by COX-1 protein and nuclear protein synthesis represented by SDH-A protein after 5-day treatment. The levels of COX-1 (•), SDH-A (♦), and ATP (■) are curve fitted to solid, dash, and dotted lines, respectively.

**Supplementary Table S1: RNA primer and templates used in mitochondrial RNA polymerase single nucleotide incorporation assay.**

| Oligonucleotides | Sequences[a] |
|---|---|
| Primer (R12) | 5'-UUUUGCCGCGCC-3' |
| Templates (D18) | 3'-CGGCGCGG**T**ACGTAAGGG-5' |
| | 3'-CGGCGCGG**G**TACTAAGGG-5' |
| | 3'-CGGCGCGG**A**CGTTAAGGG-5' |
| | 3'-CGGCGCGG**C**ATGTAAGGG-5' |

[a] The bold letter in the template sequences denotes the bases that pair with the incoming NTP.

**Supplementary References**


1. **Feng JY, Cheng G, Perry J, Barauskas O, Xu Y, Fenaux M, Eng S, Tirunagari N, Peng B, Yu M, Tian Y, Lee YJ, Stepan G, Lagpacan LL, Jin D, Hung M, Ku KS, Han B, Kitrinos K, Perron M, Birkus G, Wong KA, Zhong W, Kim CU, Carey A, Cho A, Ray AS.** 2014. Inhibition of Hepatitis C Virus Replication by GS-6620, a Potent C-Nucleoside Monophosphate Prodrug. Antimicrob Agents Chemother **58:**1930-1942.
